# *pipesnake*: generalized software for the assembly and analysis of phylogenomic datasets from conserved genomic loci

**DOI:** 10.1093/bioinformatics/btae195

**Published:** 2024-04-10

**Authors:** Ian G Brennan, Sonal Singhal, Ziad Al Bkhetan

**Affiliations:** Natural History Museum, London, SW7 5BD, United Kingdom; Division of Ecology & Evolution, The Australian National University, Canberra, ACT 2601, Australia; Department of Biology, California State University, Dominguez Hills, Carson, CA 90747, United States; Australian BioCommons, The University of Melbourne, Melbourne, VIC 3010, Australia

## Abstract

**Motivation:**

Phylogenetics has moved into the era of genomics, incorporating enormous volumes of data to study questions at both shallow and deep scales. With this increase in information, phylogeneticists need new tools and skills to manipulate and analyze these data. To facilitate these tasks and encourage reproducibility, the community is increasingly moving toward automated workflows.

**Results:**

Here we present *pipesnake*, a phylogenomics pipeline written in Nextflow for the processing, assembly, and phylogenetic estimation of genomic data from short-read sequences. *pipesnake* is an easy to use and efficient software package designed for this next era in phylogenetics.

**Availability and implementation:**

*pipesnake* is publicly available on GitHub at https://github.com/AusARG/pipesnake and accompanied by documentation and a wiki/tutorial.

## 1 Introduction

Genomic datasets employing hundreds to tens of thousands of individual markers have become common across phylogenetics, helping to resolve questions from intra-specific to inter-class scales. These projects typically target genomic loci which can be reliably identified, aligned, and modeled, and are sufficiently conserved that they remain useful at varied phylogenetic depths. Popular examples of these marker sets include Anchored Hybrid Enrichment (AHE—Lemmon *et al.* 2012) and Ultra Conserved Elements (UCE—Faircloth *et al.* 2012), however, new marker sets are being designed regularly for use at both broad and narrow foci (Johnson *et al.* 2019—Angiosperms353; Hutter *et al.* 2022—FrogCap; Hughes *et al.* 2018—FishLife). The primary impediment to developing these resources is the necessary bioinformatics architecture to execute downstream steps after data generation, including: raw read filtering and trimming, sequence read assembly, contig mapping, orthology assignment, target sequence collation, alignment and alignment processing (quality assessment, filtering, trimming), locus-specific phylogenetic estimation, and species tree estimation. With the notable exception of PHYLUCE (Faircloth 2016)—which provided the inspiration for this work—most solutions are bespoke, proprietary, or poorly documented. Many limitations to the broader use of such analysis workflows are due to clade or marker set specifics, or software installations that make reuse onerous. Here we introduce the software package *pipesnake* which has been designed to handle varied data and target types, and executed in Python and Nextflow computer languages for simplicity of data handling.

## 2 The *pipesnake* workflow

**Figure 1.**
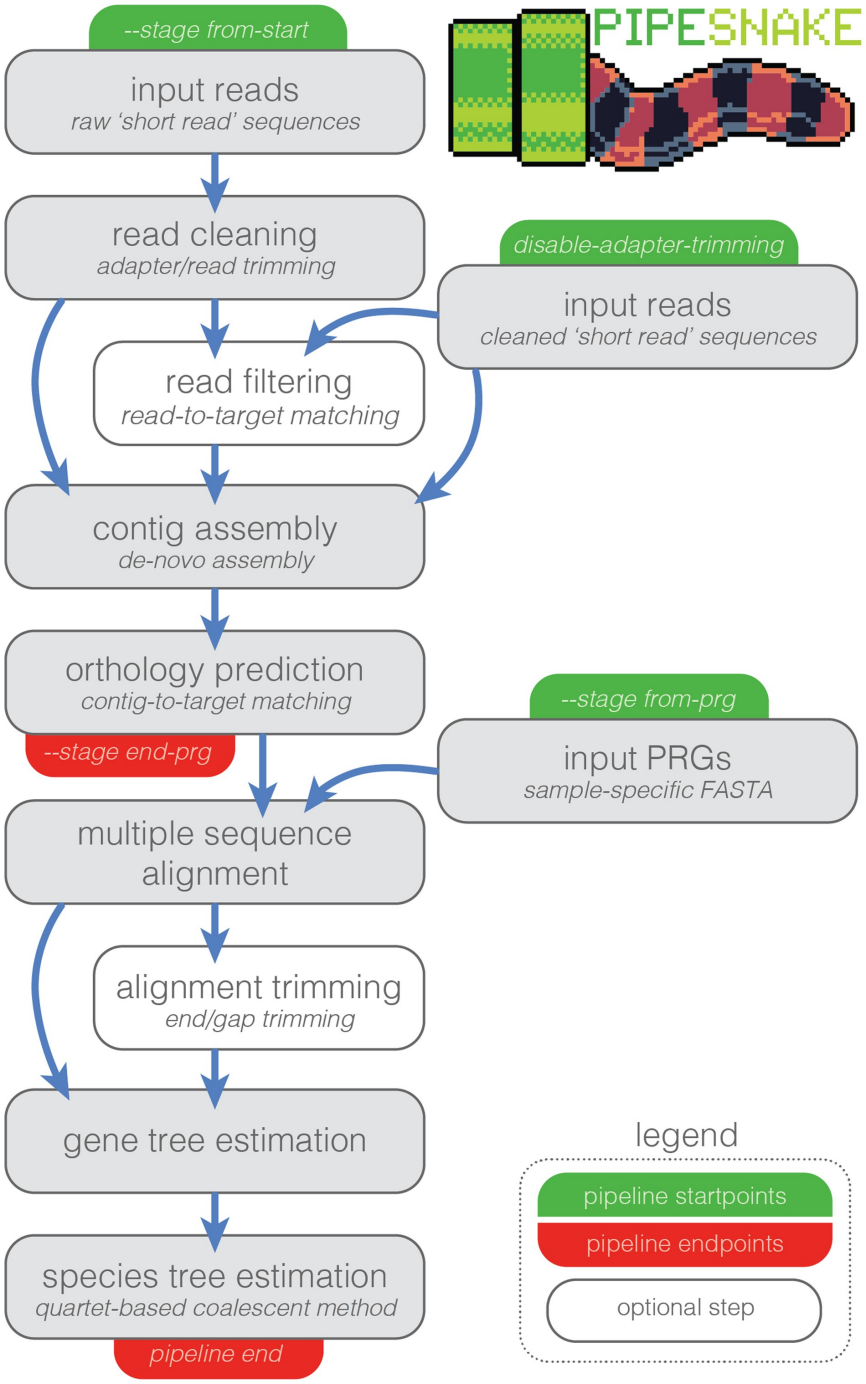
Simplified schematic of pipesnake workflow highlighting the various starting and stopping points and order of pipeline processes including optional steps. See legend in lower right.

*pipesnake* is a workflow intended to take a batch of data from second-generation short-read sequences to a species tree estimate while retaining valuable intermediate files. In the most basic form, the user provides a comma-separated sample input file, a fasta file of target loci, and raw sequence read files. From the sample info file *pipesnake* identifies all reads corresponding to a specific sample (forward and reverse), identifies and concatenates reads from multiple lanes (if applicable), and passes them to BBMAP (Bushnell 2014) to remove duplicate reads. Deduplicated reads are then submitted to TRIMMOMATIC (Bolger *et al.* 2014) for residual adapter and barcode removal and set into read pairs using PEAR (Zhang *et al.* 2014). Trimmed and paired reads are then optionally passed to BBMAP for mapping against phylogenetically informed target sequences to remove off-target reads prior to assembly (which may otherwise slow the assembly process). *pipesnake* relies on TRINITY (Grabherr *et al.* 2011) or SPAdes (Prjibelski *et al.* 2020) for contig assembly, after which contigs are mapped to target sequences via BLAT (Kent 2002) via a reciprocal search. Highest quality contig-to-target matches are then extracted and pulled into a sample-specific fasta file (which we call a Pseudo-Reference Genome—PRG). The program then pulls each target locus into a marker-specific raw alignment before passing these files along to MAFFT (Katoh and Standley 2013) for initial alignment. Alignments can then be optionally refined and trimmed using GBLOCKS (Talavera and Castresana 2007). Final alignments are then used as input for phylogenetic estimation using the preferred software (RAxML—Stamatakis 2014; or IQTREE—Minh *et al.* 2020). Locus trees are collated and used as input for ASTRAL (Zhang and Mirarab 2022) to estimate a species tree. A simplified diagram of the pipeline is included in Fig. 1.

At each step the *pipesnake* workflow generates a number of output files that are stored in process-specific directories. This allows the user to store and inspect intermediate files such as individual sample PRGs, alignment files, and locus trees. The modularity of the workflow means that if interrupted, rerunning *pipesnake* simply picks up where it left off (-resume), without the need to begin from scratch, and without the need to specify which step to begin at. Modularity also means that modifying the workflow is relatively straightforward. For example if a user would prefer to assemble contigs with software other than SPAdes or TRINITY, this requires only creating a new module file to pull a container of the software and specifying the process, outlining default parameters under docs/usage.md file, and fitting the new module into the workflow chronology in the primary pipesnake.nf file.

## 3 Motivation and example

Squamate reptiles are the largest living non-fish vertebrate clade (11 500+ species), spread widely across the globe from deserts to jungles, mountain peaks to oceanic islands, and nearly everywhere in-between. In addition to their incredible species richness and morphological diversity, squamate systematics have been an early focus for phylogenomics (Townsend *et al.* 2008, Wiens *et al.* 2010, Mulcahy *et al.* 2012). Dozens of empirical studies have used UCEs and AHE to investigate the squamate tree of life at both shallow and deep scales (Brandley *et al.* 2015, Streicher and Wiens 2017, Burbrink *et al.* 2020). To unify these marker sets Singhal *et al.* (2017) developed the Squamate Conserved Loci kit (SqCL) which incorporates >5400 genomic targets (∼5k UCEs, ∼400 AHE, ∼40 ‘legacy’ exons). Until now, the SqCL marker set has not had a well-documented reproducible workflow for assembling and analyzing new data. The lack of a user-friendly workflow has potentially acted as a limitation towards adopting this marker set, and so we present a reliable and consistent approach here. We note *pipesnake* is not, however, limited to assembling and analyzing SqCL data. The pipeline can also be comfortably used to assemble target loci from other organismal groups and for other short-read sequence data such as transcriptomes and genomes.

## 4 Implementation, flexibility, reproducibility

*pipesnake* is written in Nextflow which allows a flexible and easily customizable workflow execution on various compute infrastructures via Docker, Singularity or Conda packages. Our motivation for building *pipesnake* in Nextflow was so that we could easily provide support for local and HPC based executions. This allows *pipesnake* to interact seamlessly with workload managers like SLURM to optimize performance and juggle individual jobs. On initial use the workflow pulls necessary dependencies from online resources such as the Galaxy repository, Quay, and Bioconda. On future uses the workflow checks for locally cached software containers, excluding the need for manual local installation of dependencies. To take advantage of available resources, memory and CPU usage can be adapted by the user (see conf/base.config) and passed to pipesnake (e.g. -profile or -c) to optimize efficiency.

Regarding flexibility, many elements of the workflow's behavior can be fine-tuned using a common syntax (--[process]_args) followed by process-specific arguments between two quotes. For example, specifying a given substitution model for IQTREE (e.g.--iqtree_args “-m GTR”) is trivial and additional process-specific arguments can be strung together. Parameter defaults are stored in the conf/base.config file and specifics about their application and usage can be found in docs/usage.md. To facilitate the synthesis of separate phylogenomics projects the *pipesnake* workflow can be initiated from the alignment-formation step using the—stage command (e.g.--stage from-prg). In this instance the user provides a comma-separated sample input file and paths to PRGs of interest in fasta format, avoiding the need to reassemble samples from raw data and eliminating computational burden.

To encourage transparent and reproducible methods, *pipesnake* generates a pipeline information file that stores the versions of all software used, in addition to reports on memory and CPU usage, Nextflow commands executed, specified parameters, and a complete log file. This design means the full pipeline can be run from a single command and rerun under the same or new parameters easily. We include an example dataset of four samples which under default parameters runs from raw sequence reads to an output species tree in just a matter of minutes on a local desktop machine. Instructions for using *pipesnake*—from installation to running it on your own data—are available in the wiki/tutorial at https://github.com/AusARG/pipesnake/wiki.

## 5 Performance evaluation

To provide evidence of performance, we test the pipeline on a dataset of 9 samples with gzip compressed paired-end fastq files with an average size of 208 MB (min is 1.3 MB and max is 624 MB). Read files comprise 150-bp paired-end reads generated from SqCL hybrid enrichment across 5400 targets of varied length. Details on the required resources to run this workflow are reported in Supplementary Fig. S1 for memory and time, respectively. In this example, the assembler TRINITY is the bottleneck when it comes to the performance time required to finish the analysis. TRINITY and BBMAP deduplication require more memory than other processes. However, the required resources are within the computing power available to many genomics researchers.

## Supplementary Material

btae195_Supplementary_Data
