# Supplementary figures and images for: pipesnake: generalized software for the assembly and analysis of phylogenomic datasets from conserved genomic loci
Source: Bioinformatics. 2024 Apr 10;40(5):btae195. doi: 10.1093/bioinformatics/btae195 (PMC11082421; doi:10.1093/bioinformatics/btae195)

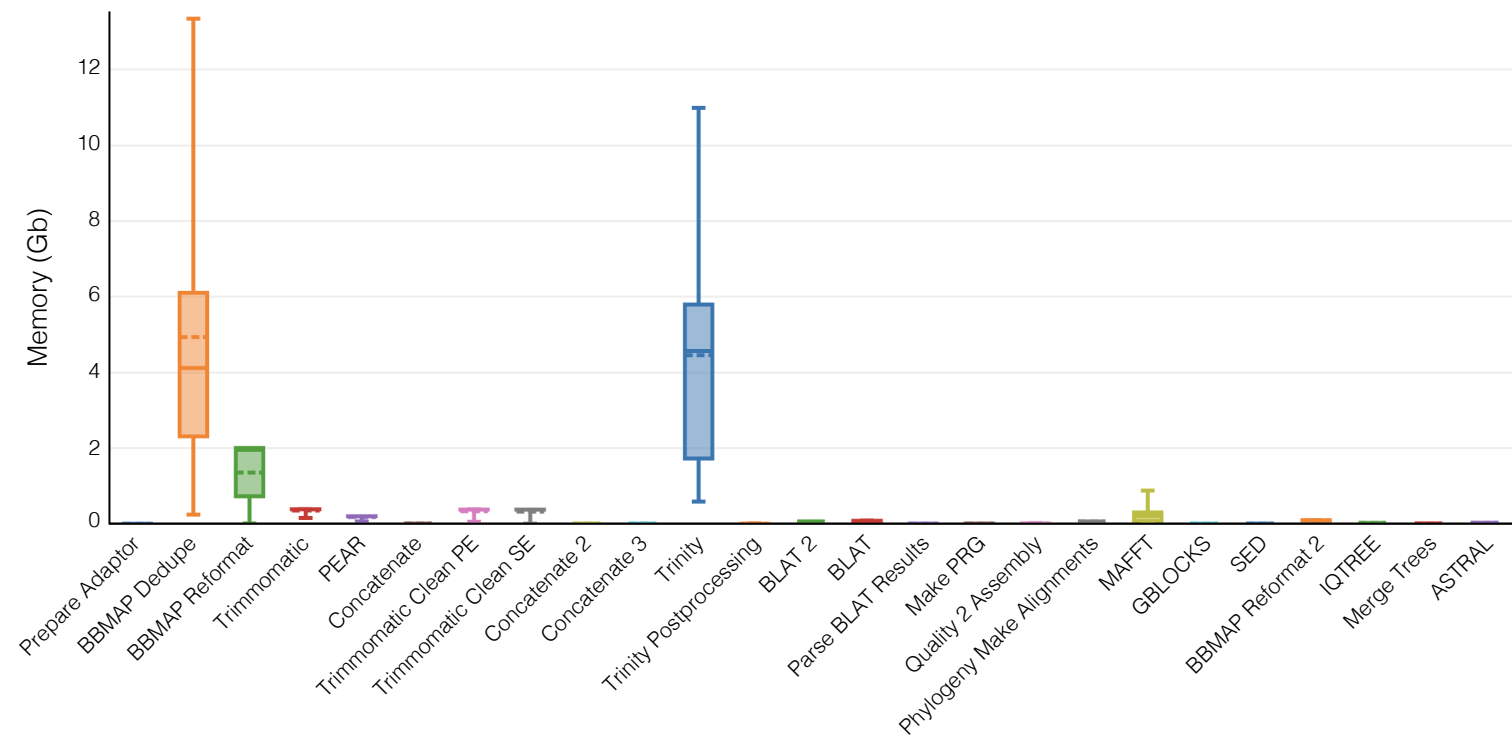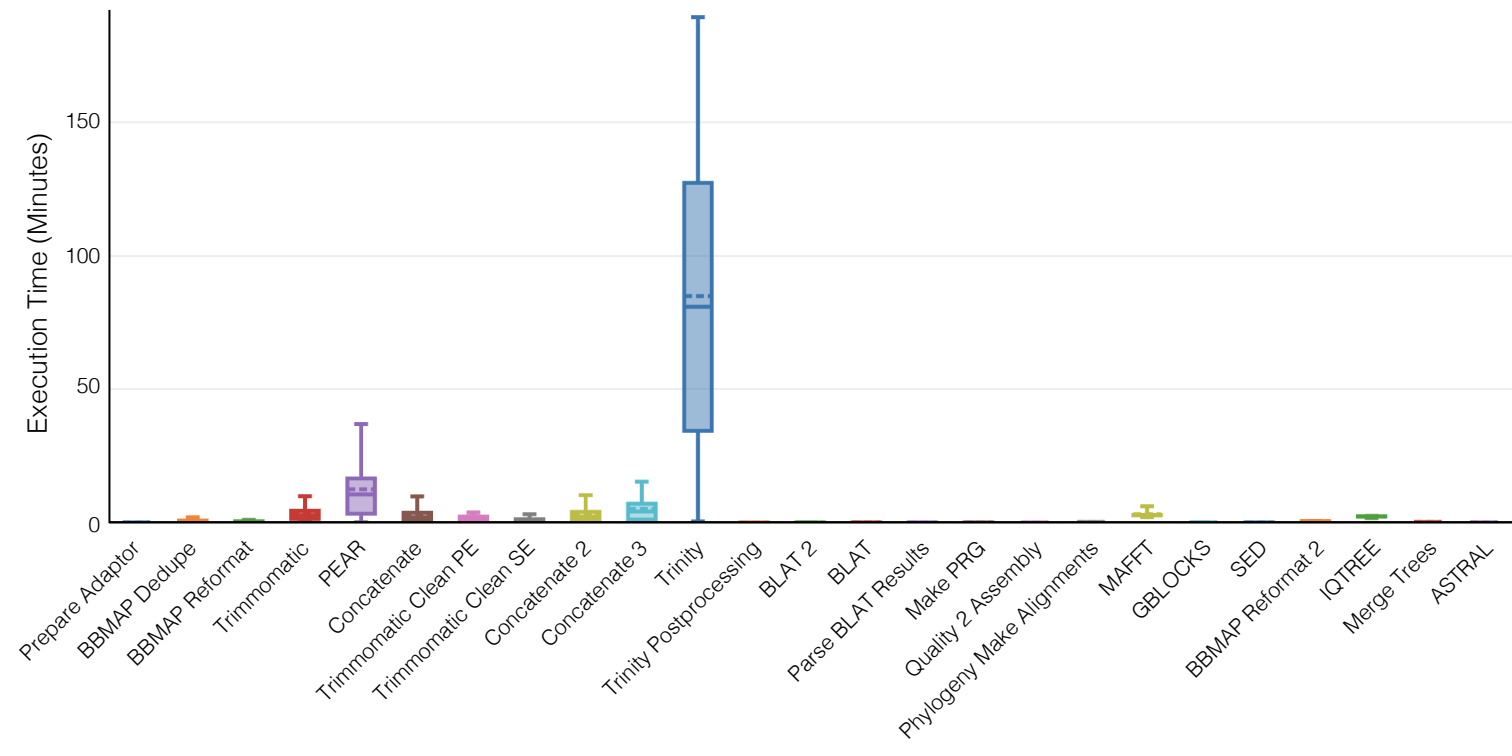

Supplement: btae195_Supplementary_Data [file btae195_supplementary_data.pdf]
